# Prognostic values, ceRNA network, and immune regulation function of SDPR in *KRAS*-mutant lung cancer

**DOI:** 10.1186/s12935-021-01756-8

**Published:** 2021-01-12

**Authors:** Xiaoqing Luo, Shunli Peng, Sijie Ding, Qin Zeng, Rong Wang, Yueyun Ma, ShiYu Chen, Yanxia Wang, Wei Wang

**Affiliations:** Department of Radiation Oncology, Nanfang Hospital, Southern Medical University, Guangzhou, 510515 People’s Republic of China

**Keywords:** SDPR (*CAVIN*2), *KRAS* mutation, Lung cancer, PD-L1, Immunotherapy

## Abstract

**Background:**

Serum Deprivation Protein Response (SDPR) plays an important role in formation of pulmonary alveoli. However, the functions and values of SDPR in lung cancer remain unknown. We explored prognostic value, expression pattern, and biological function of SDPR in non-small cell lung cancer (NSCLC) and *KRAS*-mutant lung cancers.

**Methods:**

SDPR expression was evaluated by quantitative real-time PCR (RT-qPCR), immunohistochemistry (IHC), and Western blot on human NSCLC cells, lung adenocarcinoma tissue array, *KRAS*-mutant transgenic mice, TCGA and GEO datasets. Prognostic values of SDPR were evaluated by Kaplan–Meier and Cox regression analysis. Bioinformatics implications of SDPR including SDPR-combined transcription factors (TFs) and microRNAs were predicted. In addition, correlations between SDPR, immune checkpoint molecules, and tumor infiltration models were illustrated.

**Results:**

SDPR expression was downregulated in tumor cells and tissues. Low SDPR expression was an independent factor that correlated with shorter overall survival of patients both in lung cancer and *KRAS*-mutant subgroups. Meanwhile, ceRNA network was constructed to clarify the regulatory and biological functions of SDPR. Negative correlations were found between SDPR and immune checkpoint molecules (PD-L1, TNFRSF18, TNFRSF9, and TDO2). Moreover, diversity immune infiltration models were observed in NSCLC with different SDPR expression and copy number variation (CNV) patterns.

**Conclusions:**

This study elucidated regulation network of SDPR in *KRAS*-mutant NSCLC, and it illustrated correlations between low SDPR expression and suppressed immune system, unfolding a prognostic factor and potential target for the treatment of lung cancer, especially for *KRAS*-mutant NSCLC.

## Background

Lung cancer is the most common and lethal cancer among all cancer types [1]. With the conception of individualized therapy [2], significant progress has been made based on specific pathologic subtype and molecular aberrations (e.g., epidermal growth factor receptor [EGFR], anaplastic lymphoma kinase [ALK] [3]. Kirsten rat sarcoma viral oncogene homolog (*KRAS*) mutation is frequently detected in lung adenocarcinoma and closely related with smoking status [4–6]. Several researches show that *KRAS* mutation is the most common genetic alteration type, and it occurs in approximately 10–25% of lung cancer in Western and Asia countries [7–9]. The effective clinical strategies of EGFR [10], ALK [11], and rearranged during transfection [RET] [12] aberrations remains to be explored for tumors with *KRAS* mutations [2]. The *RAS* gene family encodes a small hydrolyzed guanosine triphosphate GTPase membrane-bound protein, which interacts with downstream effectors to activate transduction of cellular survival signals, such as RAF-MEK-ERK, PI3K-AKT-mTOR, and RALGDS-RA [13–15]. Frequent mutant RAS subtypes include *KRAS* (86%), neuroblastoma rat sarcoma viral oncogene homolog *(NRAS)* (11%), and Harvey rat sarcoma viral oncogene homolog *(HRAS)* (3%) [16]. In case of NSCLC, *KRAS* mutations occur predominantly in codons 12 and 13, and most frequent variants include G12C, G12V, and G12D [9, 17].

Recently, a series of compounds targeting *KRAS*-G12C variant have been developed and achieved promising effects in preclinical experiments and phase I clinical trials [18–20]. However, it is not clear whether *KRAS* mutation, especially G12V and G12D variant, can have any clinical benefits. Meanwhile, patients with co-occurring *TP53*/*KRAS* mutations showed remarkable clinical response to immune checkpoint inhibitors (CPI) [21]. Moreover, patients with *KRAS* mutation had favorable clinical benefits of anti-PD-1/PD-L1 immunotherapy [22], and high PD-L1 expression in tumor cells was associated with improved overall survival in *KRAS* mutant patients [23]. However, the loss of STK11/LKB1 promoted programmed PD-1/PD-L1 inhibitor resistance [24]. These studies indicated that the presence of co-occurring genetic events and the mutant *KRAS* allelic content increase biological heterogeneities of *KRAS*-mutant NSCLC, which complicates the treatment of *KRAS*-mutant lung cancers.

To investigate the gene expression signature in *KRAS*-oncogene-driven lung cancer, we compared the differences between *KRAS*-mutant tumors and normal lung tissue derived from a genetically engineered mouse model (GEMM), based on expression profiling and comprehensive bioinformatics analysis. Several differentially expressed genes (DEG) were screened according to the gene expression profile, but SDPR was the only DEG that decreased in both GEMM tumors.

SDPR (also known as *CAVIN*2, NC_000002.12, gene ID 8436), a key substrate for protein kinase C, was found to play a critical role in inducing membrane curvature and participating in the formation of *caveolae* [25]. It has been reported that SDPR is a potential diagnostic indicator in cancers such as hepatocellular carcinoma and gastric cancer [26–28]. However, it remains unknown whether SDPR could be a predictor or target for lung cancer, especially in *KRAS*-mutant group. Moreover, SDPR is considered a suppressor gene in papillary thyroid cancer [29], but the regulatory mechanism of SDPR remains to be illustrated. Meanwhile, the connection between SDPR and tumor microenvironment (TME) has rarely been explored. Our study explored the gene signature, regulation, and effect of SDPR on tumor and immune infiltration, based on comprehensive bioinformatics analysis, evaluation of lung cancer specimens, and preclinical experiments.

## Methods

### Cell lines and reagents

Human non-small cell lung cancer cells (HCC4006, H23, H358, SK-LU-1 and H1299) were purchased from American Type Culture Collection (ATCC), Virginia., America. Human embryonal lung cell (MRC-5) was purchased from the Type Culture Collection of the Chinese Academy of Sciences, Shanghai, China [30]. HCC4006, H23, H358 and H1299 cells were maintained in RPMI 1640 supplemented medium, MRC-5 cells were maintained in Dulbecco’s Modified Eagle’s Medium (DMEM), and SK-LU-1 cell lines was maintained in Minimum Essential Medium (MEM), respectively. All cells were cultured in standard environment as descried previously [31].

### Transgenic mouse and establishment of *KRAS*-mutant lung cancer models

The LSL-*KRAS* mice (B6.129S4-*KRAS*tm4Tyj/JNju) were purchased from Nanjing biomedical institution of Nanjing University, Nanjing, China, and housed in specific pathogen-free (SPF) institution of Experimental animal center in Southern Medical University, Guangzhou, China. Cre recombinase induced Adeno-associated viruses (AAV-CMV-bGloin-Cre) were purchase from Shanghai genechem Co., Ltd., China. AAV-CMV-bGloin-Cre virus was in tracheally instilled into LSL-*KRAS* mice to induce *KRAS*-oncogene expression [32]. After further 4–6 months, visible tumor nodules were observed in lung tissue. Finally, tumor-bearing mice were sacrificed, and tumor tissue and normal lung tissue were collected.

### Reverse transcription, quantitative real-time PCR and Western blot

Reverse transcription, quantitative real-time PCR and Western blot were performed as described previously [33]. Oligonucleotide primers used for detection of human-SDPR, human-GAPDH (internal control), human-DACH1 and mouse-DACH1 were as follows: human-SDPR: 5′-CTCCGACGCAACCATTT-3′(sense); 5′-CTTTCTTGAGGCTATCCACTT-3′ (antisense); human-GAPDH: 5′-AGAAGGTGGGGCTCATTTG-3′ (sense); 5′-AGGGGCCATCCACAGTCTTC-3′ (antisense); human-DACH1: 5′-GGAATGGATTGTGGCTGAAC-3′ (sense); 5′-GGTATTGGACTGGTACATCAAG-3′ (antisense); mouse-DACH1: 5′-AGTGGTGGTTCTTGGGATAAGG-3′ (sense); 5′-TGAGAGGATGGCTAACTGGAA-3′ (antisense) [34]. All the reactions were performed in triplicate for each sample. Cycle threshold (Ct) values of SDPR cDNA were normalized to GAPDH using the −2ΔΔCt method. Western blot was performed according to standard protocols. These antibodies were used: SDPR (Proteintech, #12339-1-AP; RRID:2183305), β-Actin (CST, #8457; RRID:10950489). All the experiments were repeated three times.

### Clinical Specimens and Immunohistochemistry (IHC) staining

Tissue microarray with clinical pathological data of lung cancer (HLugA180Su06) was purchased from Shanghai Outdo Biotech Biotechnology Co., Ltd., China. This lung cancer microarray (HLugA180Su06) contains 94 tumors and 86 paired adjacent normal tissues. All the tissues were collected from lung adenocarcinoma patients who underwent surgical resection from July 2004 to June 2009. The follow-up was from August 2014 and ranged from 5 to 10 years. IHC staining were performed as described in Additional file 1: S1 [31]. SDPR expression were detected with a rabbit polyclonal antibody against SDPR (Proteintech, #12339-1-AP; RRID: 2183305).

### Screening of differentially expressed genes (DEGs) and identification of the abundance of tumor immune infiltration

In this study, GSE18784, GSE49200, GSE72094 and GSE48414 were downloaded from GEO dataset, and datasets (PanCancer Atlas) contained lung adenocarcinoma expression profiles and paired normal tissues were downloaded from TCGA database through cBioPortal and sangerbox download tools. “EdgeR” R package in R version 3.6.2 (The R Foundation for Statistical Computing, Vienna, Austria; https://www.r-project.org/) was used to screen out the murine DEGs between normal and tumor tissues. “CIBERSORT.R” R package was used to explore the abundance of tumor immune infiltrations in *KRAS*-mutant lung adenocarcinomas, and TIMER 1.0 and 2.0 (Tumor Immune Estimation Resource, https://cistrome.shinyapps.io/timer/) were used to identify the abundance of immune cells, such as B cells, CD4^+^ T cells, CD8^+^ T cells, Neutrophils, Macrophages and Dendritic cells at different SDPR copy number variation (CNV) patterns. The description of the above datasets and analysis processing method were described in Additional file 1: S2.

### Phylogenetic analysis of SDPR

SDPR (NC_000002.12, gene ID 8436), also known as *CAVIN*2 is located in Chromosome 2. Homo sapiens amino acid sequences of *CAV* and *CAVIN* family members were downloaded from Uniprot database. Subsequently MEGA–X (https://www.megasoftware.net/) was used to conduct sequence alignment and infer phylogenetic trees (Additional file 1: S3). The phylogeny was inferred using the Neighbor-Joining method, and the tree is created and conducted using Interactive Tree Of Life (iTOL, https://itol.embl.de/).

### Bioinformatics mining of SDPR

The information of chromosome location site and gene structure of SDPR gene were analyzed through GeneCards (https://www.genecards.org/). Protein sequences among *CAVIN* and *CAV* family members were downloaded from Uniprot database (https://www.uniprot.org/). The sequence alignment was performed to analyze the identity between *Homo sapiens*, *Mus musculus*, *Rattus norvegicus*, *Pan troglodytes*, *Macaca mulatta*, *Sus scrofa* and *Felis catus*.

GEO dataset (GSE72094) and TCGA datasets (lung adenocarcinoma, PanCancer Atlas) and Gene Expression Profiling Interactive Analysis (GEPIA) database (https://gepia.cancer-pku.cn/) were used to calculate the correlations between SDPR and Transcription factors (TFs), and evaluate overall survival (OS) of lung adenocarcinoma patients, under different SDPR expression levels and *KRAS* mutation status. TFs of SDPR were predicted using GeneCards (https://www.genecards.org/) and Promo (http://alggen.lsi.upc.es/cgi-bin/promo_v3/promo/promoinit.cgi?dirDB=TF_8.3), and microRNAs were predicted using miRanda (http://www.microrna.org/), miRDB (http://www.mirdb.org/) and TargetScan (http://www.targetscan.org/vert_72/). The potential ceRNA network of SDPR in *KRAS*-mutant lung cancer was constructed using Cytoskype software. DAVID (https://david.ncifcrf.gov/) and Gene Set Enrichment Analysis (GSEA,http://software.broadinstitute.org/gsea/register.jsp) were used to perform the Gene Ontology (GO) enrichment analysis for biological process (BP), cellular component (CC), molecule function (MF) and pathways. “pheatmap” and “ggplot2” packages were used to visualize heatmaps and bubble charts.

### Statistical analysis

All the data were analyzed by SPSS, version 20, IBM Corp., Armonk, USA. SDPR or DACH1 expression was presented as the mean ± standard deviation (SD). Bars indicate SD. * P < 0.05; ** P < 0.01. Differences between the means were examined by student’s t test or one-way analysis of variance (ANOVA). Multiple comparisons among the groups were performed using LSD method. Nonparametric test was used to analyze the SDPR scores in lung and tumor tissues, and correlation analysis was assessed by Pearson or Spearman correlation method. Kaplan–Meier method and Cox proportional hazard regression model were used to evaluate the prognostic value of SDPR in lung adenocarcinoma. Differences with a value of P < 0.05 were considered statistically significant. All of the experiments were performed at least thrice.

## Results

### The discovery of gene expression signature in *KRAS*-oncogene-driven lung cancer

**Fig. 1:**
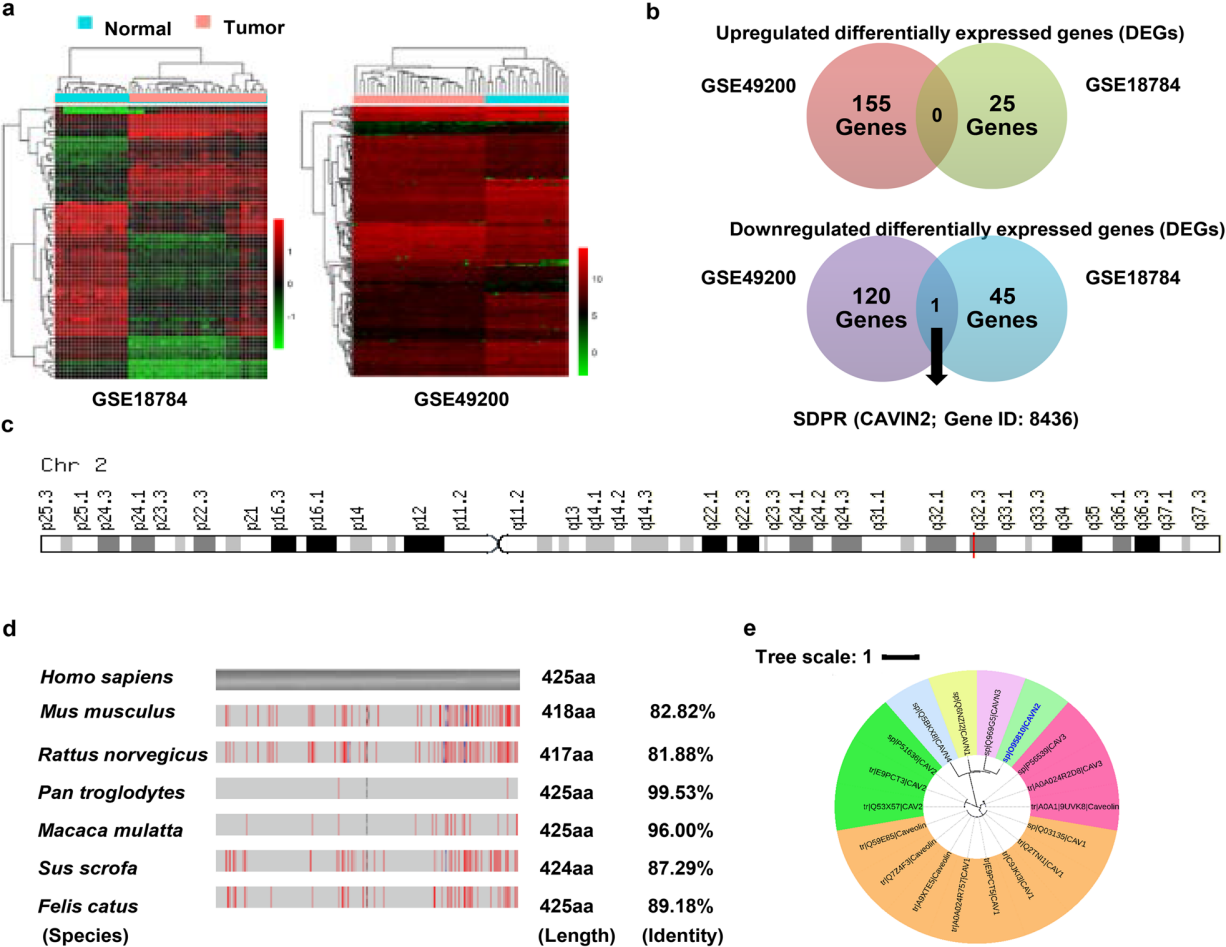
SDPR as a potential target for the treatment of *KRAS*-mutant lung cancer. **a** Transcriptional expression profiling in murine normal lung and tumor tissues with *KRAS* mutation based on GEO dataset (GSE18784, GSE49200). **b** The differentially expressed genes (DEGs) between murine normal and *KRAS*-mutant lung tumor tissues (normal vs tumor = 18 vs 34; normal vs tumor = 19 vs 31, respectively). The gene structure and phylogenetic conservative analysis of SDPR. **c** Chromosome location of SDPR is marked in red. **d** Alignment analysis results of SDPR protein among *Homo sapiens* and *other species* is shown, and different sequences are marked in red. **e** Phylogenetic tree of Homo sapiens *CAV* and *CAVIN* family proteins including SDPR (highlighted in red frame). The tree was drawn to scale based on the above family proteins sequence (Uniprotdatabase), with branch lengths in the same units as those of the evolutionary distances used to infer the phylogenetic tree. The evolutionary distances were computed and visualized using MEGA software and iTOL website (https://itol.embl.de/)

To uncover specific gene expression signature of *KRAS*-oncogene-driven lung cancer, we analyzed transcriptional expression profiles of normal lung tissues and *KRAS*-mutant lung tumor tissues based on GEO datasets (GSE18784, GSE49200), respectively, and identified differentially expressed genes (DEGs) with statistical difference (P < 0.05) between normal and tumor tissues. As shown in Fig. 1a, b, 25 upregulated DEGs and 45 downregulated DEGs were screened out based on GSE18784 dataset, using “EdgeR” R package. Using the same method, 155 upregulated DEGs and 120 downregulated DEGs were screened out based on GSE49200 dataset. The signatures between the two DEGs sets were different, indicating the heterogeneity of *KRAS*-driven tumors. Interestingly, SDPR was the only DEG that decreased in *KRAS*-mutant tumor tissues based on GEO datasets (GSE18784, GSE49200), which suggested that the downregulation of SDPR might be a specific signature during the development of *KRAS*-mutant lung cancer.

### Structure and phylogenetic conservative analysis of SDPR

SDPR, also named *CAVIN*2, is a member of *CAVIN* family, which is located at chromosome 2, q32.3 (Fig. 1c). The structures of SDPR gene include 5’UTR exon, two exons, 3′UTR exon, and one intron. Protein sequences were compared to explore conservation of SDPR during molecule and species evolution, and the alignment results showed that *Homo sapiens* SDPR shared 82.82%, 81.88%, 99.53%, 96%, 87.29% and 89.18% identity with *Mus musculus*, *Rattus norvegicus*, *Pan troglodytes*, *Macaca mulatta*, *Sus scrofa,* and *Felis catus*, respectively, which indicates that SDPR is highly conserved in mammals (Fig. 1d).

*CAV* and *CAVIN* family members play important roles in the formation and stability of pulmonary alveoli [35]. Moreover, *CAVIN* members could regulate the expression of *CAV* members. Thus, we analyzed the phylogenetic conservation of *CAV* and *CAVIN* family members. As shown in Fig. 1e, *CAV* and *CAVIN* family members are divided into two major clusters, and *CAVIN*2 shares a closer evolutionary relationship with *CAVIN*3, compared with *CAVIN*1 and *CAVIN*4.

### SDPR is downregulated in human lung adenocarcinoma, including *KRAS*-mutant group

**Fig. 2:**
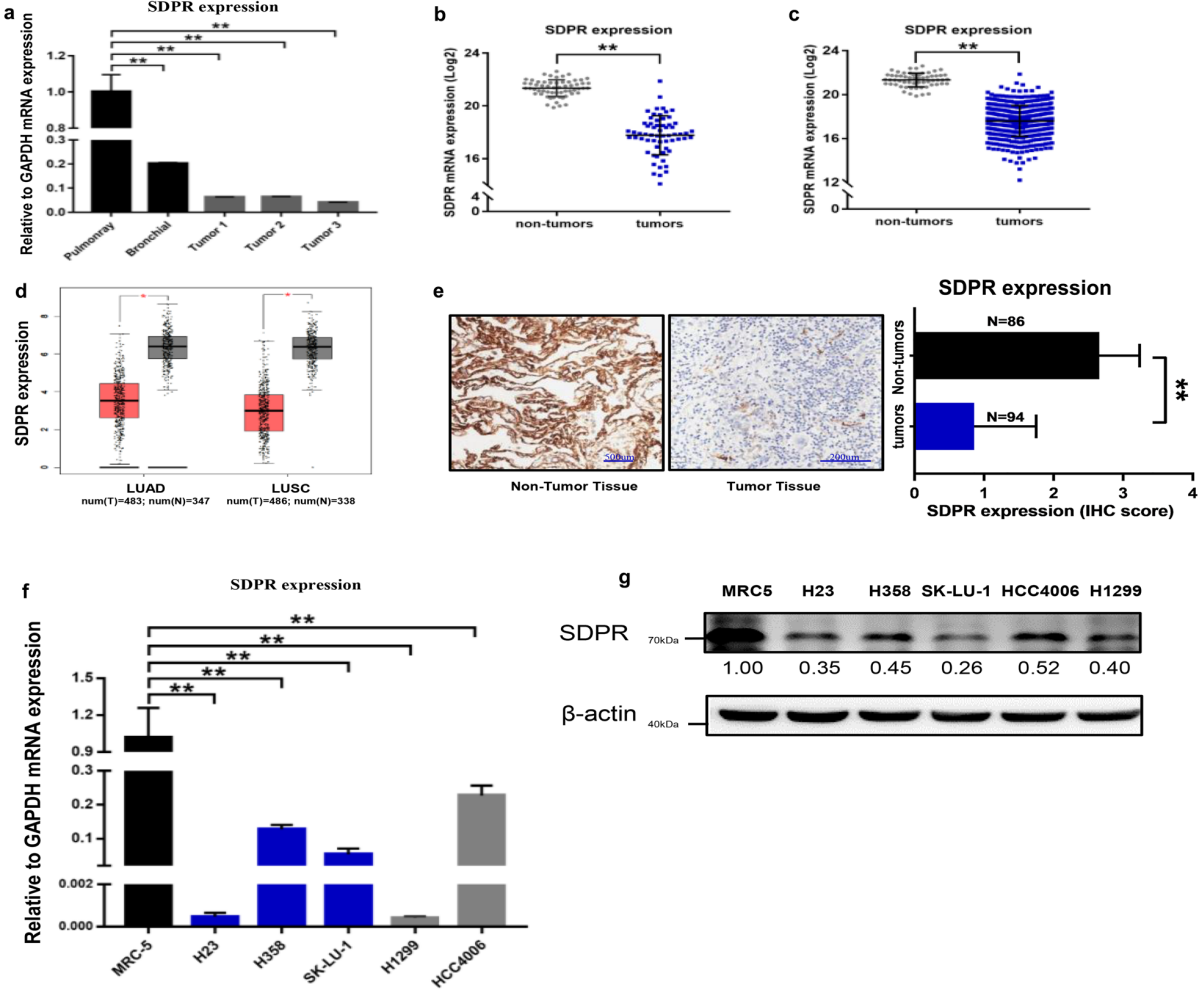
SDPR expression level is downregulated in lung cancers as well as in *KRAS*-mutant group. **a** SDPR expression in murine normal lung tissue and *KRAS*-mutant tumors measured by RT-qPCR. **b** SDPR expression level in tumor and normal tissue in the *KRAS*-mutant subgroup based on TCGA database. **c** SDPR expression level in lung tumor and normal tissue based on TCGA database. **d** SDPR expression level in lung tumor and normal tissue based on GEPIA. **e** SDPR expression in 180 lung tumor and normal tissue measured by immunohistochemistry (IHC). **f**, **g** SDPR expression in human embryonal lung cells, *KRAS*-mutant and *KRAS* wild-type lung cancer cells measured by RT-qPCR and western blot, and the results of western blot were quantified using Image J quantitative analysis software. Bars indicate SD. *P < 0.05; **P < 0.01. All of the experiments were repeated three times

To identify the SDPR expression level in mouse and human lung tissues and tumors, we established *KRAS*-oncogene-driven lung cancer models [32] and detected SDPR expression using RT-qPCR. As shown in Fig. 2a, higher SDPR expression was detected in pulmonary than in bronchial tissue. Moreover, lower SDPR expression was observed in *KRAS*-mutant tumor tissues (P < 0.05). We further confirmed SDPR expression in human tissues and found a similar result in *KRAS*-mutant tumors. As shown in Fig. 2b–e, SDPR expression significantly decreased in *KRAS*-mutant specimens as well as all lung tumors compared with normal tissue (P < 0.05). In addition, low SDPR expression was detected in *KRAS*-mutant and *KRAS*-wild type NSCLC cell lines compared with immortalized normal lung cells, MRC-5 (Fig. 2f–g).

### Low expression of SDPR is associated with a poor prognosis in NSCLC patients

**Fig. 3:**
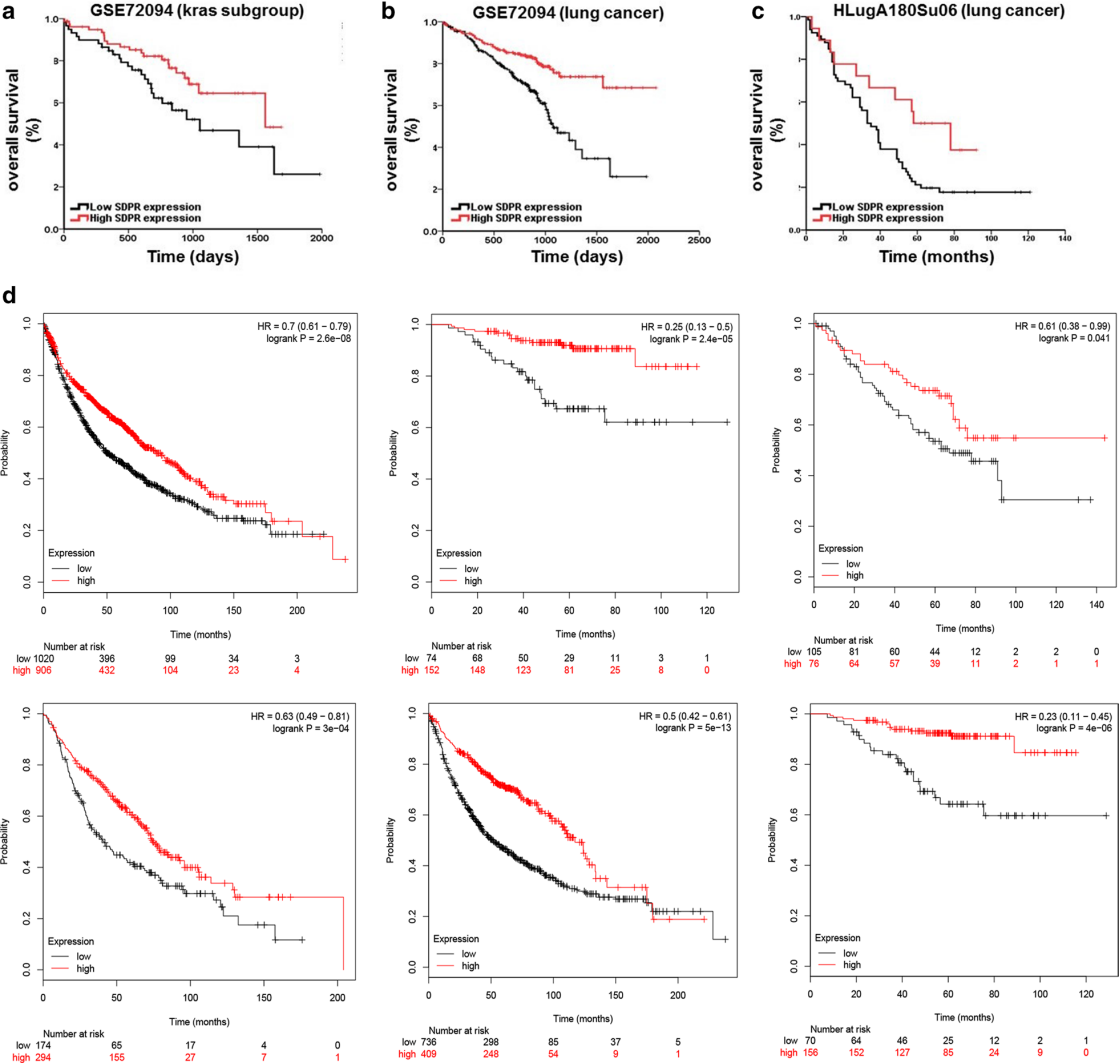
Prognostic values of SDPR in lung adenocarcinoma, as well as in *KRAS*-mutant subtype. **a**, **b** Impact of SDPR expression on the overall survival in *KRAS*-mutant and all of the lung adenocarcinoma patients based on GEO dataset (GSE72094). **c** Impact of SDPR expression on the overall survival in lung adenocarcinoma patients based on lung cancer microarray (HLugA180Su06). **d** Impact of SDPR expression on the overall survival in lung adenocarcinoma patients based on the cohorts downloaded from GEPIA

As shown in Fig. 3a–c, low expression of SDPR was associated with shorter OS in NSCLC patients as well as in *KRAS*-mutant group, based on GEO dataset and lung cancer microarray (GSE72094, HLugA180Su06, P < 0.05). Similar results were found in NSCLC patients using GEPIA (Fig. 3d, P < 0.05). Meanwhile, univariate survival analysis indicated that low SDPR expression was associated with the shorter OS in NSCLC patients as well as in *KRAS*-mutant group (*KRAS*-mutant lung adenocarcinoma, P < 0.05, hazard ratio [HR] = 0.7; lung adenocarcinoma, P < 0.05, hazard ratio [HR] = 0.7; Table 1). Moreover, multivariate survival analysis showed that SDPR expression and stage were independent predictors of prognosis in lung adenocarcinoma patients as well as in *KRAS*-mutant group (Table 1). These data highlight the prognostic value of SDPR in human lung adenocarcinoma, especially in *KRAS*-mutant subgroup.

**Table 1:** Impact of SDPR expression and clinic pathologic characteristics in lung adenocarcinoma

Clinicopathologic variable^a^	HR	95% CI	P value
a. SDPR expression associations with overall survival in *KRAS*-mutant patients (GSE72094) using Cox regression			
Expression (low vs high)	0.55	0.31–0.98	0.04

Clinicopathologic variable^b^	HR	95% CI	P value
b. Multivariate survival model in *KRAS*-mutant patients (GSE72094) using Cox regression			
SDPR expression (low vs high)	0.53	0.29–0.96	0.04
Gender	0.77	0.41–1.45	0.42
Smoking	0.75	0.37–1.51	0.42
Pathological stage (I vs II–IV)	2.05	1.13–3.70	0.02
c. Impaction of SDPR and clinicopathologic characteristics on overall survival			
			

Clinicopathologic variable^a^	HR	95% CI	P value
d. SDPR expression associations with overall survival in lung cancer patients (GSE72094) using Cox regression			
SDPR expression (low vs high)	0.44	0.30–0.64	< 0.001

Clinicopathologic variable^b^	HR	95% CI	P value
e. Multivariate survival model using Cox regression			
SDPR expression (low vs high)	0.47	0.32–0.70	< 0.001
Gender	0.55	0.38–0.82	< 0.001
Smoking	0.78	0.503–1.21	0.26
Pathological stage			< 0.001
Stage I	0.75	0.16–3.54	0.71
Stage II	0.25	0.12–0.54	< 0.001
Stage III	0.48	0.21–1.07	0.07
Stage IV	0.82	0.37–1.83	0.63
f. Impaction of SDPR and clinicopathologic characteristics on overall survival			

### Construction of competing endogenous RNA (ceRNA) network of SDPR in *KRAS*-mutant lung adenocarcinoma pathway

**Fig. 4:**
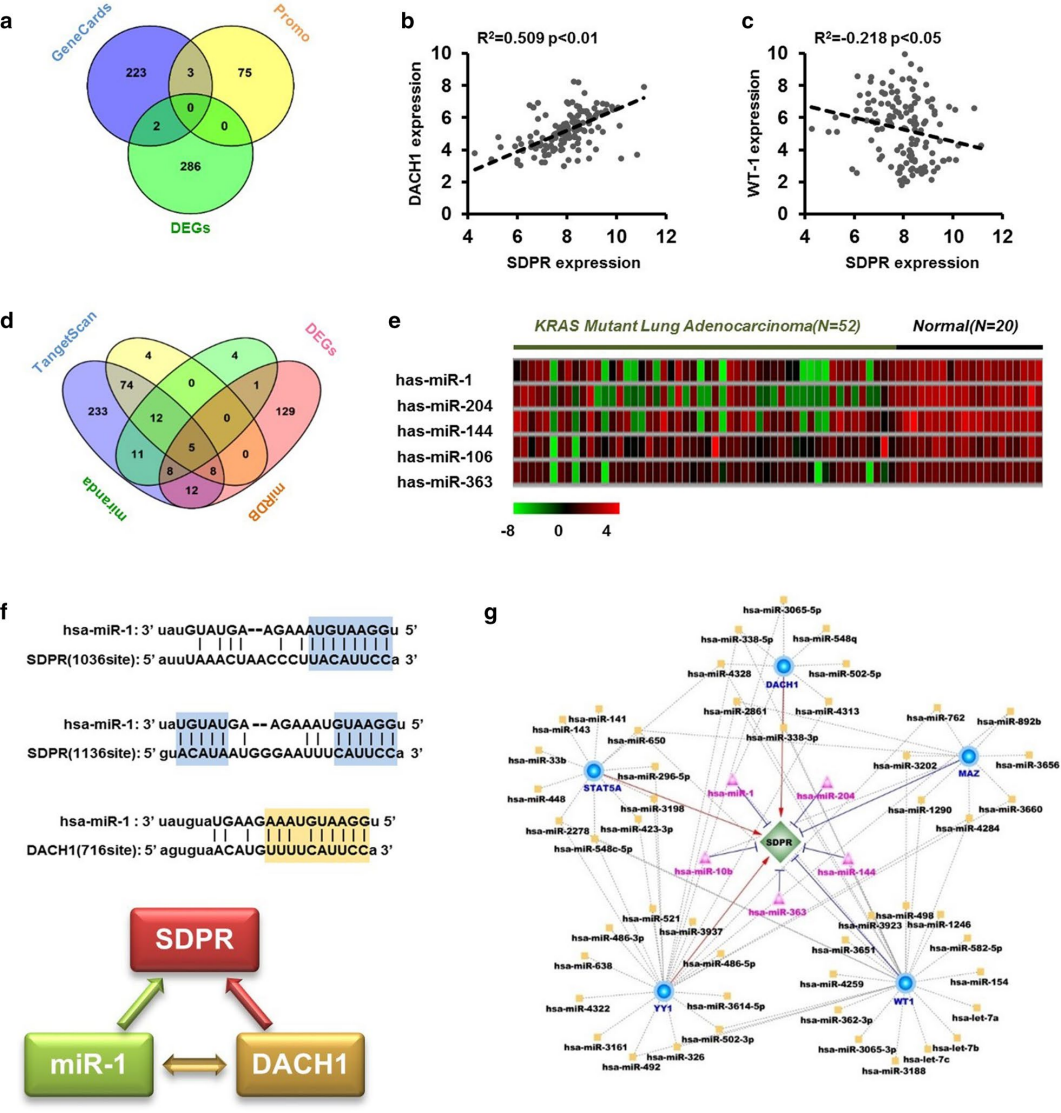
Construction of ceRNA network of SDPR in *KRAS*-mutant lung adenocarcinoma. **a** Prediction of upstream transcription factors (TFs) of SDPR in *KRAS*-mutant lung adenocarcinoma. *KRAS*-mutant specific TFs were screened out based on GEO dataset (GSE72094), and intersected with SDPR related TFs predicted by online database (Promo or Genecards). **b** The correlations between SDPR and two predicted TFs, DACH1 and WT-1 based on TCGA dataset (Atlas). **c**, **d** Prediction of upstream miRNAs of SDPR in *KRAS*-mutant lung adenocarcinoma. *KRAS*-mutant specific miRNAs were screened out based on TCGA dataset (GSE48414), then intersected with SDPR combined miRNAs predicted by miRDB, miranda and targetScan. Five miRNAs finally were screened out and the expression levels in normal tissue and *KRAS*-mutant lung carcinoma were shown with heat map. **f** Complementary sequences between hsa-miR-1, DACH1 and SDPR. **g** Construction of ceRNA network to visualize the regulation models of SDPR, TFs and miRNA in *KRAS*-mutant lung carcinoma

To identify the upstream regulatory structure of SDPR in *KRAS*-mutant lung cancer, DEGs based on GSE72094 and three public predicted websites (TargetScan, miRDB and miranda) were used (Fig. 4a). Briefly, 139 expression profiles of *KRAS*-mutant patients with complete clinical information were collected (GSE72094), and DEGs sets between low and high SDPR group were screened out using “EdgeR” R package. Three public websites, TargetScan, miRDB and miranda, were used to predict potential combinations between SDPR and transcription factors. As shown in Fig. 4a, two transcription factors (DACH1, WT-1) were identified based on DEGs and TargetScan websites. Moreover, SDPR correlated positively with DACH1 (R^2^ = 0.509, P < 0.01; Fig. 4b) and negatively with WT-1 (R^2^ = − 0.218, P < 0.05; Fig. 4c). We detected the expression of DACH1 in NSCLC cell lines, MRC5 cells and the *KRAS*-oncogene-driven lung cancer mice. The DACH1 expression in bronchial tissue was lower than that in normal lung tissue based on *KRAS* oncogenic mice models. Meanwhile, DACH1 expression was lower in tumor tissue than in normal lung tissue. Moreover, The DACH1 expression in NSCLC cells was lower than that in MRC5 cells (Additional file 1: Figure S1).

Similar to the above screening method of transcription factors, a set of miRNAs was predicted, and five miRNAs (hsa-miR-1, hsa-miR-204, hsa-miR-144, hsa-miR-105 and hsa-miR-363) were ultimately screened out, which were observed in the above 4 miRNA sets (Fig. 4d). All of them were downregulated in *KRAS*-mutant lung adenocarcinoma compared with normal lung tissues (Fig. 4e). Interestingly, we found some potential complementary sequences between hsa-miR-1 and DACH-1 (Fig. 4f), indicating that the above miRNAs and TFs may form a complex network to regulate SDPR expression. Thus, we screened a series of miRNAs with potential combination sequence with SDPR-related TFs, and constructed a competing endogenous RNA (ceRNA) network of SDPR in *KRAS*-mutant lung adenocarcinoma (Fig. 4g).

### Biological enrichment analysis of SDPR downstream pathway

**Fig. 5:**
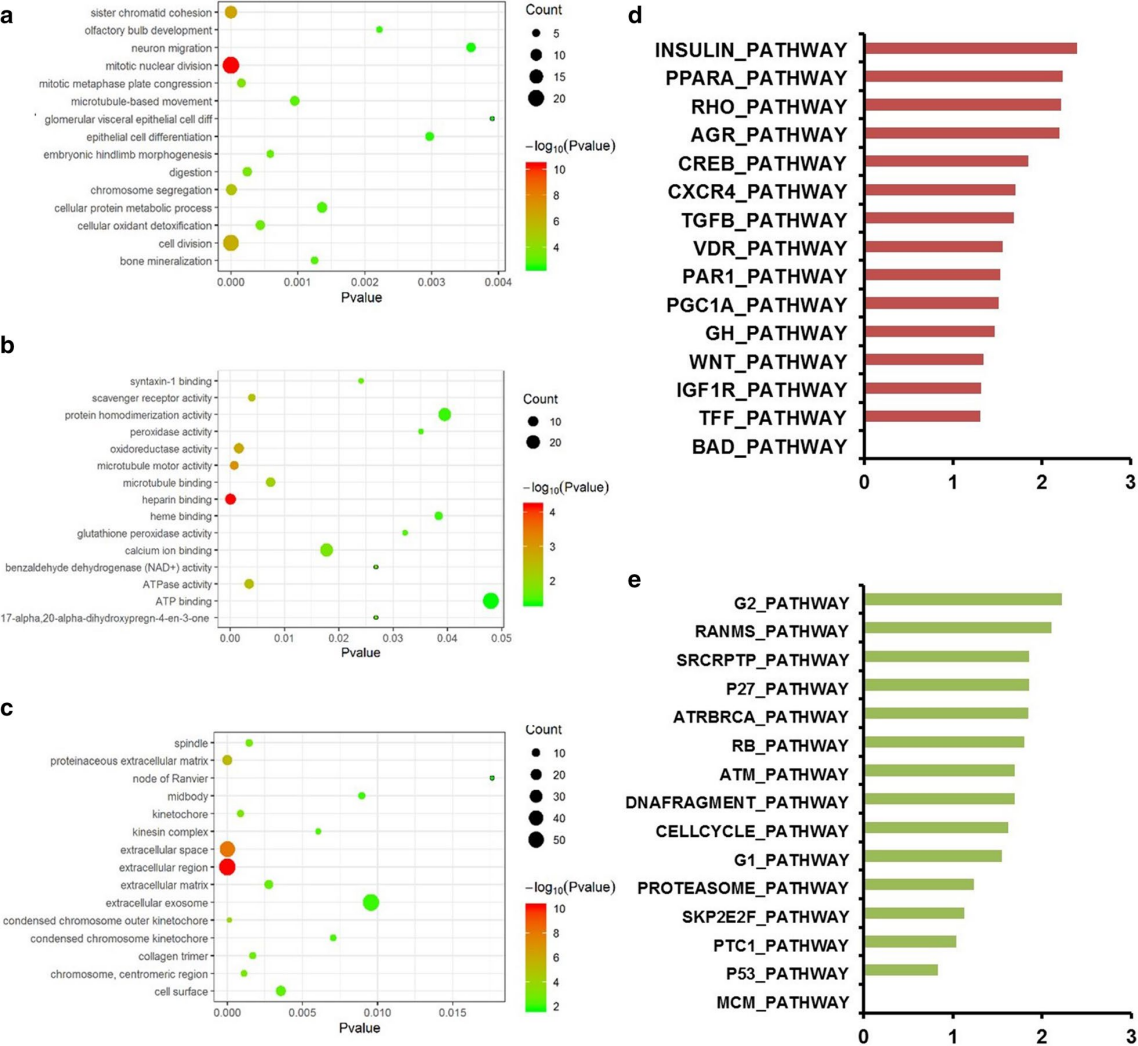
Biological enrichment analysis of SDPR downstream pathway based on GEO dataset (GSE72094). **a**–**c** Annotation of the biological function of biological process, cellular component and molecule function using DAVID online software. **d**, **e** Prediction of SDPR-related pathways by enrichment plots from gene set enrichment analysis (GSEA)

To explore the downstream pathway of SDPR, DEGs based on GSE72094 were explored to identify biological differences between tissues with low and high SDPR expression in *KRAS*-mutant lung cancer. Gene ontology analysis was performed using DAVID online software to unfold the biological function of biological process, cellular component and molecule function among the above DEGs. As shown in Fig. 5a–c, biological processes were mainly associated with cell mitosis and cell cycle, and the differences of cellular components were mainly located in the extracellular space, exosomes, and matrix. In addition, there were a series of members related to redox balance and energy transfer, indicating the close interaction between SDPR expression and metabolism. Moreover, GSEA analysis results showed that G2 pathway and TGF-beta pathway were most likely associated with the above DEGs (Fig. 5d, e).

### Correlation between SDPR, immune negative regulatory molecules and immune infiltration models

Recently, SDPR was reported to play an important role in cancer progression and metastasis via epithelial mesenchymal transition (EMT) in gastric and breast cancers [27, 36]. However, the function of SDPR in lung cancer, especially in *KRAS*-mutant group, remains unclear. Since different SDPR expression levels are accompanied with changes in extracellular components (Fig. 5c), we hypothesized that SDPR expression may be closely related with tumor environment. Thus, we explored the correlation between SDPR, immune checkpoint molecules and immune infiltration models.

**Fig. 6:**
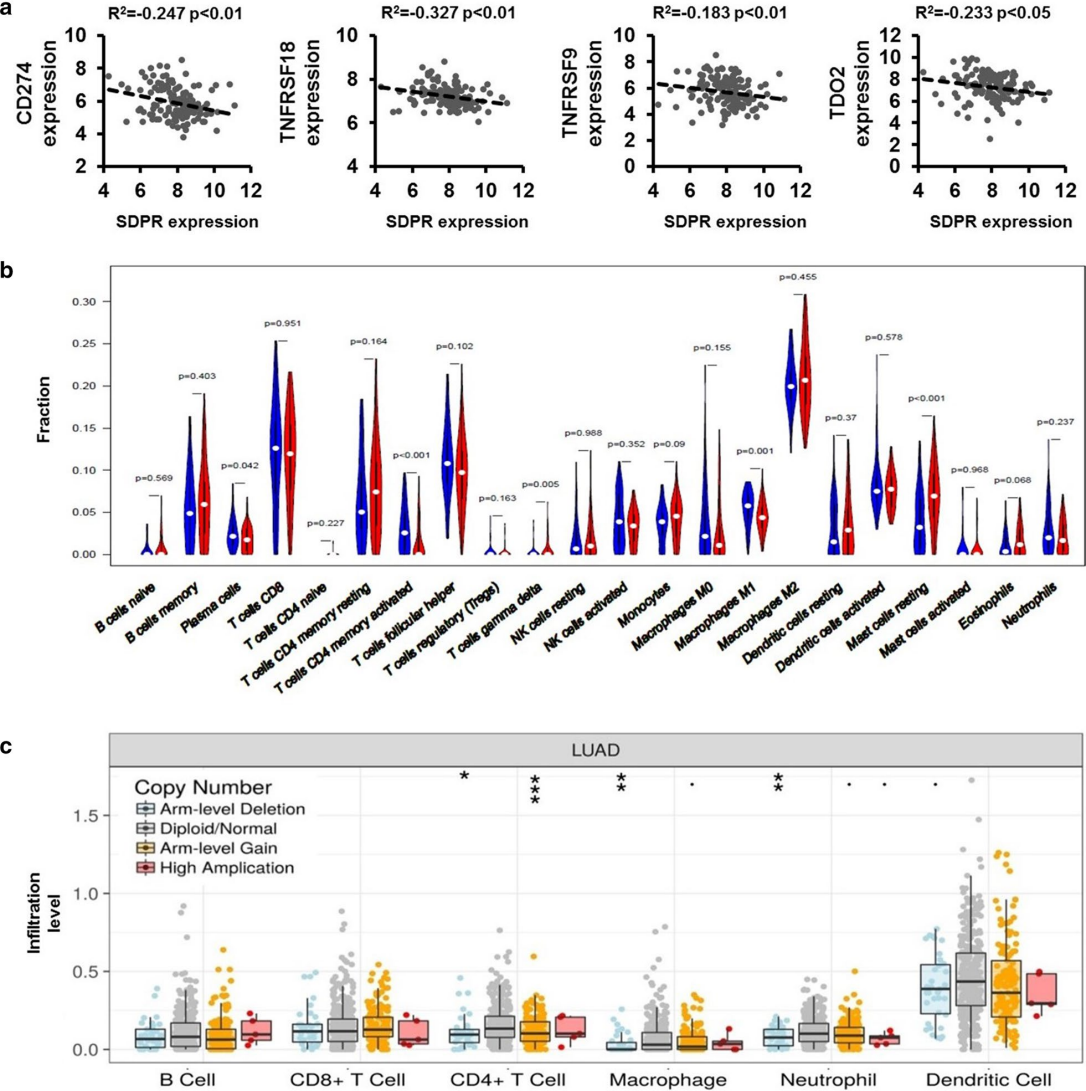
Correlation between SDPR, immune checkpoint molecules and abundance of tumor immune infiltrations. **a** Correlations between SDPR expression and immune checkpoint molecules based on GEO dataset (GSE72094). **b** Abundance of immune infiltrates in *KRAS*-mutant lung cancer with different somatic CNV patterns of SDPR based on GEO dataset (GSE72094). Low SDPR expression group marked in blue, and high SDPR expression group marked in red. **c** Correlations between SDPR mRNA expression and abundance of immune infiltrates in lung adenocarcinoma using TIMER (comprehensive resource for the clinical relevance of tumor-immune infiltrations) system. **d** Abundance of immune infiltrates in lung adenocarcinoma with different somatic CNV patterns of SDPR using TIMER system

As shown in Fig. 6a, SDPR expression level correlated negatively with PD-L1(CD274), GITR(TNFRSF18), 4-1BBR(TNFRSF9) and TDO2 (R^2^ = − 0.247, − 0.327, − 0.183, − 0.233, respectively; P < 0.05). Since the role of SDPR in immune infiltration is unclear, we analyzed the abundance of immune cells in lung cancers at different SDPR expression levels and copy number variation (CNVs) patterns. In *KRAS*-mutant subgroups, cancer tissue with lower expression of SDPR was accompanied with less infiltration of γ T cells and resting mast cells but higher abundance of plasma cells, CD4^+^ memory activated T cells and M1 macrophages (Fig. 6b). Meanwhile, SDPR expression in lung adenocarcinoma correlated positively with infiltration of memory B cells, endothelial cells, M1 and M2 macrophages, myeloid dendritic cells, neutrophils, memory resting CD4^+^ T cells, CD8^+^ T cells, but correlated negatively with M0 macrophages, plasma B cells, and CD4^+^ memory activated T cells based on TIMER 2.0 website (Table 2). In addition, lung adenocarcinoma with SDPR arm-level deletion showed less infiltration of CD4^+^ T cells, macrophages and neutrophils in TME (Fig. 6c).

**Table 2:** Correlation between SDPR expression and immune infiltration in lung adenocarcinoma

Infiltrates	rho	p	adj.p
B cell memory_CIBERSORT	0.14	0.00	0.00
B cell memory_CIBERSORT-ABS	0.18	0.00	0.00
B cell plasma_XCELL	*− 0.21*	0.00	0.00
B cell_EPIC	0.10	0.03	0.07
B cell_MCPCOUNTER	0.15	0.00	0.00
B cell_QUANTISEQ	0.12	0.01	0.02
Cancer associated fibroblast_EPIC	*− 0.09*	0.04	0.10
Cancer associated fibroblast_XCELL	0.36	0.00	0.00
Common lymphoid progenitor_XCELL	*− 0.21*	0.00	0.00
Common myeloid progenitor_XCELL	0.17	0.00	0.00
Endothelial cell_EPIC	0.51	0.00	0.00
Endothelial cell_MCPCOUNTER	0.61	0.00	0.00
Endothelial cell_XCELL	0.47	0.00	0.00
Eosinophil_XCELL	0.15	0.00	0.00
Granulocyte-monocyte progenitor_XCELL	0.39	0.00	0.00
Hematopoietic stem cell_XCELL	0.62	0.00	0.00
Macrophage M0_CIBERSORT	*− 0.33*	0.00	0.00
Macrophage M0_CIBERSORT-ABS	*− 0.22*	0.00	0.00
Macrophage M1_CIBERSORT	*− 0.12*	0.01	0.03
Macrophage M1_QUANTISEQ	0.24	0.00	0.00
Macrophage M2_CIBERSORT	0.22	0.00	0.00
Macrophage M2_CIBERSORT-ABS	0.38	0.00	0.00
Macrophage M2_QUANTISEQ	0.40	0.00	0.00
Macrophage M2_XCELL	0.29	0.00	0.00
Macrophage_EPIC	0.14	0.00	0.01
Macrophage_TIMER	0.19	0.00	0.00
Macrophage_XCELL	0.10	0.03	0.07
Mast cell activated_CIBERSORT	0.38	0.00	0.00
Mast cell activated_CIBERSORT-ABS	0.42	0.00	0.00
Mast cell resting_CIBERSORT	− *0.23*	0.00	0.00
Mast cell resting_CIBERSORT-ABS	− *0.20*	0.00	0.00
Mast cell_XCELL	0.32	0.00	0.00
MDSC_TIDE	− *0.50*	0.00	0.00
Monocyte_CIBERSORT	0.34	0.00	0.00
Monocyte_CIBERSORT-ABS	0.40	0.00	0.00
Monocyte_QUANTISEQ	− *0.22*	0.00	0.00
Monocyte_XCELL	0.18	0.00	0.00
Myeloid dendritic cell activated_CIBERSORT	0.13	0.00	0.01
Myeloid dendritic cell activated_CIBERSORT-ABS	0.17	0.00	0.00
Myeloid dendritic cell activated_XCELL	0.15	0.00	0.00
Myeloid dendritic cell resting_CIBERSORT	0.13	0.00	0.01
Myeloid dendritic cell resting_CIBERSORT-ABS	0.17	0.00	0.00
Myeloid dendritic cell_MCPCOUNTER	0.29	0.00	0.00
Myeloid dendritic cell_QUANTISEQ	− *0.19*	0.00	0.00
Myeloid dendritic cell_TIMER	0.17	0.00	0.00
Myeloid dendritic cell_XCELL	0.29	0.00	0.00
Neutrophil_MCPCOUNTER	0.33	0.00	0.00
Neutrophil_QUANTISEQ	0.18	0.00	0.00
Neutrophil_TIMER	0.11	0.01	0.03
NK cell activated_CIBERSORT-ABS	0.09	0.04	0.10
NK cell_EPIC	− *0.10*	0.03	0.07
Plasmacytoid dendritic cell_XCELL	− *0.15*	0.00	0.00
T cell CD4 + (non-regulatory)_XCELL	0.09	0.04	0.08
T cell CD4 + effector memory_XCELL	0.12	0.01	0.02
T cell CD4 + memory activated_CIBERSORT	− *0.24*	0.00	0.00
T cell CD4 + memory activated_CIBERSORT-ABS	− *0.23*	0.00	0.00
T cell CD4 + memory resting_CIBERSORT	0.28	0.00	0.00
T cell CD4 + memory resting_CIBERSORT-ABS	0.37	0.00	0.00
T cell CD4 + Th1_XCELL	− *0.38*	0.00	0.00
T cell CD4 + Th2_XCELL	− *0.38*	0.00	0.00
T cell CD4 + _EPIC	0.30	0.00	0.00
T cell CD8 + naive_XCELL	− *0.21*	0.00	0.00
T cell CD8 + _CIBERSORT-ABS	0.16	0.00	0.00
T cell CD8 + _EPIC	0.24	0.00	0.00
T cell CD8 + _TIMER	0.17	0.00	0.00
T cell follicular helper_CIBERSORT	− *0.11*	0.01	0.03
T cell regulatory (Tregs)_CIBERSORT	− *0.17*	0.00	0.00
T cell regulatory (Tregs)_QUANTISEQ	0.34	0.00	0.00

Significant correlation between immune cell subgroups and SDPR expression were shown in Table 2 based on TIMER, CIBERSORT, quanTIseq, xCell, MCP-counter and EPIC algorithms. Positive correlation was marked in underline, while negative correlation was marked in italics. P< 0.01 were marked as 0.00

These results illustrated close relationship between SDPR, PD-L1(CD274), GITR(TNFRSF18), 4-1BBR(TNFRSF9), TDO2, and abundance of immune cells in human lung adenocarcinoma, especially in *KRAS*-mutant subgroups.

## Discussion

Over 8 different variants of *KRAS* mutation have been identified at codons 12, 13 and 61 in NSCLC [37]. Several studies explored the therapeutic vulnerability and prognostic differences between the *KRAS* mutation subtypes [38–40]. However, *KRAS* mutant status may not be recommended to select NSCLC patients for specific treatment such as adjuvant chemotherapy. Meanwhile, there were no significant differences in the phosphorylation level of MEK/ERK kinase among the above variants, despite phosphorylation of AKT and activation of RAL seem to differ between *KRAS*-G12C and *KRAS*-G12V cells [4, 38]. In summary, no specific *KRAS* variants were validated as ideal prognostic factors of survival or vulnerability indicators for treatment of *KRAS*-mutant tumors. In our study, we found that SDPR expression was not only decreased in *KRAS*-mutant NSCLC cells, and *KRAS*-driven murine tumor from GEMMs, but also downregulated in human NSCLC specimens based on GEO datasets, TCGA datasets, and lung adenocarcinoma tissue array (Fig. 2a–g). Moreover, SDPR expression was suggested to be an independent prognostic factor in lung cancer (Fig. 3a–d, Table 1). Our research provides a potential target for the prognosis and treatment of NSCLC independent of *KRAS* variants. More biological experiments and clinical trials are needed to validate and complement our conclusions.

Co-occurring genetic events were frequently observed in *KRAS*-mutant lung tumors, unlike other oncogene-driven lung cancers [40, 41]. STK11 co-mutations (KL), TP53 co-mutations (KP), and CDKN2A/B inactivation plus low thyroid transcription factor-1 (TTF-1) expression (KC) were considered as classical models among *KRAS*-mutant tumors, and may induce different biological behaviors and characteristics of tumors [40, 42]. Co-occurring STK11 or KEAP1 mutations were associated with worse OS in *KRAS*-mutant NSCLCs [9, 40]. Moreover, the lowest levels of PD-L1 and deficient inflammatory immune cells infiltration were found in the KL group. In contrast, the KP group with the highest PD-L1 expression was infiltrated with active inflammation (mainly T cell inflammation) [24]. Our research found negative correlations between SDPR, PD-L1, and immune cells in *KRAS*-mutant lung cancers (Fig. 6a–c). More studies should explore the influence of co-occurring genetic events on SDPR expression and malignant biological behaviors of tumors.

Previous research explored the prognostic and diagnostic significance of SDPR in gastric cancer [27], hepatocellular carcinoma [28], and papillary thyroid cancer (PTC) [29]. Our research originally found downregulation of SDPR in lung cancers as well as in *KRAS*-mutant subgroup (Fig. 2a–g), and innovatively explored the immune checkpoint molecules and abundance of immune infiltrations at different SDPR expression and CNVs models (Fig. 6a–c, Table 2). Those results provide a novel theory for the immune regulatory functions of SDPR in tumorigenesis, progression and metastasis.

In terms of the regulation and function of SDPR in lung cancer, the reason leads to the depression of SDPR is unclear. It was reported that MiR-577 regulates TGF-β in gastric cancer through a SDPR-modulated positive-feedback loop [27]. Moreover, overexpression of SDPR inhibited the activity of ERK and NF-κB pathways in breast cancer [36]. In our study, a series of pathways, including the TGF-β pathway, were enriched between SDPR-low and SDPR-high specimens in *KRAS* mutant lung cancers (Fig. 5d). In addition, results of GO analysis indicated different distribution of extracellular components depending on SDPR expression (Fig. 5c). Our study screened out a series of TFs and miRNAs as promising candidates for the upstream targets of SDPR in *KRAS*-mutant cancers, and we constructed a ceRNA regulation network of SDPR in *KRAS* mutant lung cancers, which provided useful information for the molecule regulatory network of SDPR in *KRAS*-mutant lung cancers.

## Conclusions

In our study, a decrease of SDPR was found in lung cancers as well as in *KRAS*-mutant subgroup, and which may be a promising prognostic marker for the survival of patients with lung cancer. Moreover, systematic exploration of SDPR in gene location, species conservation, function, and potential regulatory network was illustrated in lung cancer, especially in *KRAS*-mutant tumors. In addition, our research originally unfolded the correlation between SDPR, immune checkpoint molecules, and abundance of immune infiltrations. In summary, SDPR could be a promising prognostic factor and potential target for the treatment of lung cancer, especially for *KRAS*-mutant adenocarcinomas.

## **Additional file**

**Additional file 1.** Additional materials, mehtods and figure 

## Data Availability

All data generated or analyzed during this study are included in this published article.

## Abbreviations

SDPR: Serum Deprivation Protein Response
NSCLC: Non-small cell lung cancer
IHC: Immunohistochemistry
TCGA: The Cancer Genome Atlas
TFs: Transcription factors
TNFRSF18: Tumor Necrosis Factor Receptor Superfamily Member 18
TNFRSF9: Tumor Necrosis Factor Receptor Superfamily Member 9
TDO2: Tryptophan 2,3-dioxygenase 2
TME: Tumor microenvironment
CNV: Copy number variation
EGFR: Epidermal growth factor receptor
ALK: Anaplastic lymphoma kinase
ATCC1: American Type Culture Collection
GEMM: Genetically engineered mouse model
GSEA: Gene set enrichment analysis
GEO: Gene expression omnibus
DEG: Differentially expressed gene
