# Supplementary material for: Prognostic values, ceRNA network, and immune regulation function of SDPR in KRAS-mutant lung cancer
Source: Cancer Cell Int. 2021 Jan 12;21:49. doi: 10.1186/s12935-021-01756-8 (PMC7802324; doi:10.1186/s12935-021-01756-8)
Supplement: Supplementary file 1 — Additional file 1. Additional materials, mehtods and figure [file 12935_2021_1756_MOESM1_ESM.docx]

**Prognostic values, ceRNA network, and immune regulation function of SDPR in *KRAS*-mutant lung cancer**

**Xiaoqing Luo^1^, Shunli Peng^1^, Sijie Ding^1^, Qin Zeng^1^, Rong Wang^1^, Yueyun Ma^1^, ShiYu Chen^1^, Yanxia Wang^1^, Wei Wang^1^**

**Supplementary materials and methods**

**Supplementary file S1. Immunohistochemistry (IHC) evaluation**

IHC staining were performed as described ([2](#_ENREF_2)). Positive area from 0%, 1–25%, 26–50%, 51–75% and >75% were considered as 0, 1, 2, 3, 4, respectively, and the staining intensity with weakly stained, moderately stained and strongly stained was considered as 0, 1, 2, 3, respectively. SDPR expression was detected using SDPR antibodies (12339-1-AP) and assessed by equation = positive areas score × intensity score. 0 was considered no expression (0 score), 1-3 was considered as low expression (1 score), 4-6 was considered as moderate expression (2 score), and >7 was considered as high expression (3 score).

**Supplementary file S2. Basic information of GSE18784, GSE49200, GSE72094 and GSE48414 datasets and Screening process for differentially expressed genes (DEGs) and immune infiltration models**

GSE18784 and GSE49200 datasets contains murine transcriptional expression profiles of normal lung tissue and *KRAS*-mutant lung cancer tissues. Expression data without annotation probe was removed, and the remained data were normalized using “limma” packages. Then cluster analysis was applied to divide samples into normal group and tumor group, and those samples that described as normal tissue but clustered into tumor group or described as tumor tissue but clustered into normal group were excluded. Subsequently, “EdgeR” R package in R version 3.6.2 (The R Foundation for Statistical Computing, Vienna, Austria; http://www.r-project.org/) was used to screen out the DEGs between normal murine tissues and tumor tissues, and p Value < 0.05 and |log_2_ FC| ≥ 2 were defined as the threshold for screening DEGs. Venn diagram draw by Venny 2.1.0 (http://bioinfogp.cnb.csic.es/tools/venny/index.html) was used to visualize the common and different DEGs between GSE18784 and GSE49200 datasets.

GSE72094 dataset contains transcriptional expression profiles of 442 human lung adenocarcinoma with mutation status of *KRAS*, STK11 and TP53. Expression profiles contained *KRAS*-mutant lung adenocarcinoma complete clinical information were separated (N=139) and divided into SDPR-low (N=59) and SDPR-high (N=80) group. Subsequently, the normalization and DEGs screening between low SDPR expression group and high SDPR expression group were performed according to the above method.

“CIBERSORT.R” R package were used to explore the abundance of tumor immune infiltrations in *KRAS*-mutant lung adenocarcinomas, and TIMER (Tumor Immune Estimation Resource, https://cistrome.shinyapps.io/timer/） were used to identify the abundance of B cells, CD4^+^ T cells, CD8^+^ T cells, Neutrophils, Macrophages and Dendritic cells in lung adenocarcinoma with different SDPR copy number variation (CNV) patterns.

GSE48414 dataset contains microRNA-profiles of 154 human lung adenocarcinomas and 20 paired normal lung tissues, Expression profiles contained *KRAS*-mutant lung adenocarcinoma were separated into a new matrix file, and the normalization and DEGs screening between normal tissue and *KRAS*-mutant adenocarcinomas were performed according to the above method.

**Supplementary file S3. Phylogenetic analysis of SDPR**

Homo sapiens amino acid sequences of CAV and CAVIN family members were downloaded from Uniprot database. Subsequently MEGA–X (https://www.megasoftware.net/) were used to conduct sequence alignment and infer phylogenetic trees. The phylogeny was inferred using the Neighbor-Joining method, and the percentage of replicate trees in which the associated taxa clustered together in the bootstrap test (1000 replicates) are shown next to the branches. The tree is drawn to scale, with branch lengths in the same units as those of the evolutionary distances used to infer the phylogenetic tree ([1](#_ENREF_1)). Ultimately, Newick file was created and conducted into Interactive Tree Of Life (iTOL, https://itol.embl.de/) to display, annotation and management phylogenetic tree.

**Supplementary Figure**

**Supplementary Figure S1. DACH1 expression were downregulated in NSCLC cell lines and tumor tissue of *KRAS* oncogenic mice**

1. DACH1 expression in murine normal lung tissue and *KRAS*-mutant tumors measured by RT-qPCR. b. DACH1 expression in human embryonal lung cells (MRC5), *KRAS*-mutant and *KRAS*-wide type lung cancer cells measured by RT-qPCR. DACH1 expression was presented as the mean ± standard deviation (SD), and differences between the means were examined by one-way analysis of variance (ANOVA). Multiple comparisons among the groups were performed using LSD method. Differences with a value of P < 0.05 were considered to be statistically significant. Bars indicate SD, ** P < 0.01.

**
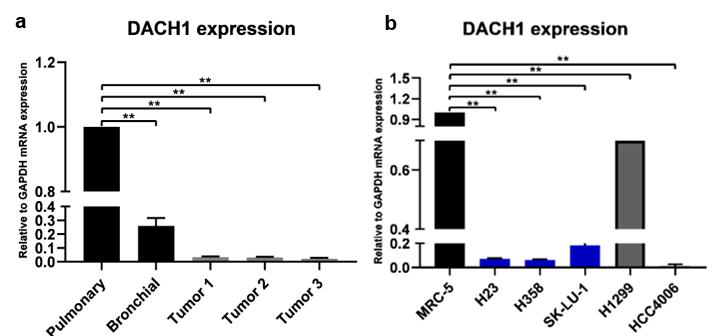
**

**Reference**

1. Yang M, Huang W, Sun Y, Liang H, Chen M, Wu X, et al. Prognosis and modulation mechanisms of COMMD6 in human tumours based on expression profiling and comprehensive bioinformatics analysis. British journal of cancer. 2019;121(8):699-709.

2. Liu T, Sun Q, Li Q, Yang H, Zhang Y, Wang R, et al. Dual PI3K/mTOR inhibitors, GSK2126458 and PKI-587, suppress tumor progression and increase radiosensitivity in nasopharyngeal carcinoma. Mol Cancer Ther. 2015;14(2):429-39.
